# Association between high sensitivity cardiac troponin and mortality risk in the non-diabetic population: findings from the National Health and Nutrition Examination Survey

**DOI:** 10.1186/s12933-023-02003-2

**Published:** 2023-10-30

**Authors:** Lin Liu, Yuen Ting Cheng, Aimin Xu, Bernard M. Y. Cheung

**Affiliations:** 1 Department of Medicine, School of Clinical Medicine, The University of Hong Kong, Hong Kong, China; 2 State Key Laboratory of Pharmaceutical Biotechnology, The University of Hong Kong, Pokfulam, Hong Kong, China; 3 Institute of Cardiovascular Science and Medicine, The University of Hong Kong, Pokfulam, Hong Kong, China; 4 Department of Medicine, University of Hong Kong, Queen Mary Hospital, Pokfulam, Hong Kong, China

**Keywords:** Troponin, Prediabetes, Cardiovascular disease risk, Mortality, Preventive cardiology

## Abstract

**Objective:**

We investigated the association of high-sensitivity cardiac troponin (Hs-cTn) with all-cause and cardiovascular mortality in non-diabetic individuals.

**Methods:**

This study included 10,393 participants without known diabetes and cardiovascular disease from the US National Health and Nutrition Examination Survey (NHANES). Serum Hs-cTnI and Hs-cTnT concentrations were measured. Prediabetes was defined as fasting blood glucose between 100 and 125 mg/dL or HbA1c between 5.7 and 6.4%. Cox proportional hazard models were used to estimate hazard ratios (HRs) and 95% confidence intervals (CIs) for mortality risk. Time-dependent receiver operating characteristics (tROC) curves were utilized to measure the predictive performance of the biomarkers. Net Reclassification Improvement (NRI) were calculated to estimate the improvement in risk classification for adding Hs-cTnT or Hs-cTnI to the standard models based on Framingham risk factors.

**Results:**

The mean age of the participants was 48.1 ± 19.1 years, with 53.3% being female and 25.8% being prediabetic. After multivariable adjustment, compared to those with Hs-cTnI concentration less than the limit of detection, the HRs (95% CIs) of the participants with Hs-cTnI concentration higher than the 99th upper reference limit were 1.74 (1.35, 2.24) for all-cause mortality and 2.10 (1.36, 3.24) for cardiovascular mortality. The corresponding HRs (95% CIs) for Hs-cTnT were 2.07 (1.53, 2.81) and 2.92 (1.47, 5.80) for all-cause and cardiovascular mortality. There was a significant interaction between prediabetes and Hs-cTnI on the mortality risk; a positive relationship was only observed in prediabetic individuals. No interaction was observed between prediabetes and Hs-cTnT on mortality risk. The Areas Under tROC indicated both Hs-cTnT and Hs-cTnI show better predictive performance in cardiovascular mortality than in all-cause mortality. NRI (95% CI) for adding Hs-cTnT to the standard model were 0.25 (0.21, 0.27) and 0.33 (0.26, 0.39) for all-cause and cardiovascular mortality. The corresponding NRI (95% CI) for Hs-cTnI were 0.04 (0, 0.06) and 0.07 (0.01, 0.13).

**Conclusions:**

Elevated blood levels of Hs-cTnI and Hs-cTnT are associated with increased mortality. Measurement of Hs-cTnT in non-diabetic subjects, particularly those with prediabetes, may help identify individuals at an increased risk of cardiovascular disease and provide early and more intensive risk factor modification.

**Supplementary Information:**

The online version contains supplementary material available at 10.1186/s12933-023-02003-2.

## Introduction

Prediabetes is characterized by higher-than-normal blood glucose or hemoglobin A1c (HbA1c) levels, resulting from underlying pathophysiologic defects including insulin resistance, alpha- and beta-cell dysfunction, and inflammation. Individuals with prediabetes are at risk of developing long-term complications associated with diabetes, including microvascular and macrovascular disorders [1]. According to the National Diabetes Statistics Report [2], nearly one-third of US adults are affected by prediabetes, and a high proportion of those affected are likely to develop diabetes, which significantly burdens the healthcare system. Consequently, the US Preventive Services Task Force recommends early screening for prediabetes in the general population [3].

Cardiac troponin (cTn) is a protein highly expressed in cardiomyocytes. The high sensitivity assays of cTn enable sensitive detection of acute myocardial infarction, making it the preferred biomarker in acute coronary syndromes diagnosis [4]. However, elevated high-sensitivity cardiac troponin (Hs-cTn) concentrations have also been found in asymptomatic individuals and are associated with increased risks of cardiovascular events and mortality [5]. Elevated Hs-cTnT levels indicative of subclinical myocardial damage have been independently related to major adverse cardiovascular events in patients with prediabetes [6]. However, the association between Hs-cTn, prediabetes, and mortality is not well understood. Therefore, this study aims to determine whether Hs-cTnI and Hs-cTnT concentrations are associated with all-cause and cardiovascular mortality in nondiabetic subjects.

## Method

### Study population

The data for this study were derived from the National Health and Nutrition Examination Survey (NHANES), a prospective cohort study conducted in the United States designed to assess the health and individuals’ nutritional status in the United States. Details of the analytic guideline have been published elsewhere [7]. NHANES was conducted by the National Center for Health Statistics of the Centers for Disease Control and Prevention (CDC) and was approved by the institutional review board of the National Center for Health Statistics. All participants provided written informed consent.

For this study, we used data from NHANES collected between 1999 and 2004, and included individuals aged 18 years or over. Diabetes was defined as fasting blood glucose (FBG) ≥ 126 mg/dL or hemoglobin A1c (HbA1c) ≥ 6.5% or the use of anti-diabetic medication [8]. Prediabetes was defined using American Diabetes Association (ADA) criteria: FBG between 100 and 125 mg/dL or HbA1c between 5.7 and 6.4%. Normoglycemia was defined as FBG less than 100 mg/dL and HbA1c less than 5.7%. History of cardiovascular disease included self-report angina, myocardial infarction, congestive heart failure, coronary heart disease, and stroke. After excluding those with diabetes at baseline or missing value for definition (N = 3799), with missing Hs-cTn measurements (N = 1472), and with a history of cardiovascular disease or missing information (N = 1364), a total of 10,393 participants were included in the analysis.

### Measurement of Hs-cTn concentration

Sera from stored surplus specimens were used for Hs-cTn measurement. The majority (93%) of stored serum samples had never undergone a prior freeze–thaw cycle. All measurements were performed during 2018–2020 at the University of Maryland School of Medicine, Baltimore, Maryland [9]. Hs-cTnI was measured using Siemens Centaur XP, with a limit of detection (LOD) of 1.6 ng/L. The sex-specific 99th percentile of the upper reference limit [99th upper reference limit (URL)] was 58 ng/L for males and 39.6 ng/L for females. Hs-cTnT was measured using Roche Cobas e601, with the LOD of 3 ng/L and the 99th URLs being 22 ng/L for males and 14 ng/L for females. In addition, other two Hs-cTnI were measured using the Abbott ARCHITECT i2000SR and the Ortho Vitros 3600, namely Hs-cTnI (A) and Hs-cTnI (O) in the current study. The LOD were 1.7 ng/L and 0.39 ng/L for Hs-cTnI (A) and Hs-cTnI (O). The sex-specific 99th URL was 35 ng/L for males and 17 ng/L for females, in terms of Hs-cTnI (A). The corresponding cutoffs were 12 ng/L and 9 ng/L for Hs-cTnI (O). All of the cutoffs were provided by the International Federation of Clinical Chemistry and Laboratory Medicine (IFCC) [10].

### Assessment of covariates

Information on age, sex, race, smoking status, alcohol drinking, and medication use (antidiabetic, antihypertensive, statin, and antiplatelet medications) was collected from structured questionnaires. Blood pressure (BP), body weight, and height were measured by trained examiners. Hypertension was defined as systolic BP ≥ 140 mmHg or diastolic BP ≥ 90 mmHg or with antihypertensive medication use [11]. The estimated glomerular filtration rate (eGFR) was calculated using the Modification of Diet in Renal Disease formula [12]. FBG, HbA1c, total cholesterol, triglyceride, high-density lipid cholesterol, and serum creatinine were measured according to a standard procedure described previously [13].

### Ascertainment of mortality

Mortality from all causes and cardiovascular disease was determined by matching to the National Death Index through 31 December 2019. The International Classification of Diseases, 10th version (ICD-10) was used to clarify the cause of death. Cardiovascular mortality was defined as death resulting from heart diseases (I00–I09, I11, I13, I20–I51) and cerebrovascular diseases (I60–I69).

### Statistical analysis

Participants were divided into 3 groups using LOD and 99th URL of Hs-cTn: Hs-cTn concentration < LOD, Hs-cTn concentration between LOD and 99th URL, and Hs-cTn concentration ≥ 99th URL. Baseline characteristics are presented as mean (continuous variables) or frequency distribution (categorical variables). One-way ANOVA or chi-square tests were applied to compare the differences among Hs-cTn categories for continuous and categorical variables. Kaplan–Meier curves and log-rank tests were performed to compare the differences in mortality among Hs-cTn categories. We used the Cox proportional hazard model to estimate the relationship between Hs-cTn concentration and all-cause and cardiovascular mortality (Hs-cTn concentration < LOD as reference). The proportional hazards assumption was satisfied based on Schoenfeld residual testing. Two regression models were fitted: model 1 adjusted for age and sex, and model 2 adjusted for age, sex, race, current smoker, systolic BP, diastolic BP, body mass index, total cholesterol, high-density lipid cholesterol, eGFR, prediabetes status, antihypertensive medication use, antiplatelet medication use, and statin use. To assess the associations between Hs-cTn concentration and prediabetes and mortality, we calculated the adjusted hazard ratios (HRs), 95% confidence intervals (95% CIs) of Hs-cTn concentration stratified by prediabetes status and further tested the interactions between Hs-cTn and prediabetes on mortality. Participants were further divided into six groups: normoglycemia with Hs-cTn concentration < LOD, normoglycemia with Hs-cTn concentration between LOD and 99th URL, normoglycemia with Hs-cTn concentration ≥ 99th URL, prediabetes with Hs-cTn concentration < LOD, prediabetes with Hs-cTn concentration between LOD and 99th URL, and prediabetes with Hs-cTn concentration ≥ 99th URL. Kaplan–Meier curves and a multivariable Cox regression model (normoglycemia with Hs-cTn concentration < LOD as reference) were also conducted. Time-dependent receiver operating characteristics (tROC) curves and area under tROC [tAUC] were used to estimate the performance of Hs-cTns in predicting all-cause and cardiovascular mortality. Furthermore, risk difference based Net Reclassification Improvement (NRI) were calculated to estimate the improvement in 10-year risk classification for incorporating Hs-cTnT or Hs-cTnI to the standard models based on Framingham risk factors. The Framingham risk factors include age, sex, smoking, systolic blood pressure, total cholesterol, high density lipid cholesterol, and anti-hypertensive medication. We did not apply the survey weights to analyses due to the highly selected of the study population and our focus on internal comparisons within the sample. All analyses mentioned above were performed using R 4.2.1 (R Foundation for Statistical Computing). A two-tailed P-value < 0.05 was considered statistically significant in all analyses.

## Results

A total of 10,393 participants (mean ± SD age was 48.06 ± 19.06 years, 5537 [53.3%] were female) were included in the study. The median [interquartile range (IQR)] concentrations of Hs-cTnI and Hs-cTnT were 2.59 ng/L [1.16–5.04 ng/L] and 5.18 ng/L [3.58–8.16 ng/L], respectively. The numbers of participants with Hs-cTn concentration < LOD, between LOD to 99th URL and ≥ 99th URL were, respectively, 3046, 6818, 169 for Hs-cTnI and 1455, 8265, 673 for Hs-cTnT. A total of 2681 (25.8%) participants were diagnosed as having prediabetes. Baseline characteristics according to Hs-cTnI concentration are shown in Table 1. Compared with participants with Hs-cTnI concentration < LOD, those with Hs-cTnI concentration ≥ 99th URL were older (mean age, 64.57 years vs. 37.32 years), less likely to be female (47.9% vs. 74.8%) and had a higher prevalence of prediabetes (39.1% vs. 15.5%) and hypertension (66.3% vs. 12.0%). Similar results (Additional file 1: Table S1) were observed for the Hs-cTnT concentration.

**Table 1:** Baseline characteristic according to Hs-cTnI concentration

	Overall	< LOD	LOD to 99th URL	≥ 99th URL	P-value
	10,393	3406	6818	169	
Age, year	48.06 ± 19.06	37.32 ± 13.50	53.01 ± 19.15	64.57 ± 16.82	< 0.001
Female (%)	5537 (53.3)	2546 (74.8)	2910 (42.7)	81 (47.9)	< 0.001
Race and ethnicity (%)					< 0.001
Hispanic	2731 (26.3)	1027 (30.2)	1657 (24.3)	47 (27.8)	
Non-Hispanic White	5522 (53.1)	1691 (49.6)	3757 (55.1)	74 (43.8)	
Non-Hispanic Black	1784 (17.2)	546 (16.0)	1190 (17.5)	48 (28.4)	
Others	356 (3.4)	142 (4.2)	214 (3.1)	0 (0.0)	
Current smoker (%)	2310 (22.2)	819 (24.0)	1464 (21.5)	27 (16.0)	0.01
Alcohol drinker (%)	6718 (64.6)	2140 (62.8)	4480 (65.7)	98 (58.0)	0.005
Systolic BP, mmHg	125.05 ± 21.01	115.11 ± 14.29	129.65 ± 21.89	139.41 ± 25.28	< 0.001
Diastolic BP, mmHg	70.56 ± 13.81	68.59 ± 11.79	71.51 ± 14.50	71.33 ± 18.21	< 0.001
Body mass index, kg/m²	27.93 ± 5.98	27.93 ± 6.29	27.94 ± 5.82	27.53 ± 5.75	0.694
Prediabetes (%)	2681 (25.8)	529 (15.5)	2086 (30.6)	66 (39.1)	< 0.001
Hypertension (%)	3256 (31.3)	410 (12.0)	2734 (40.1)	112 (66.3)	< 0.001
Antihypertensive medication (%)	1862 (17.9)	235 (6.9)	1545 (22.7)	82 (48.5)	< 0.001
Statin (%)	845 (8.1)	106 (3.1)	704 (10.3)	35 (20.7)	< 0.001
Antiplatelet medication (%)	102 (1.0)	4 (0.1)	91 (1.3)	7 (4.1)	< 0.001
Fasting blood glucose, mg/dL	95.28 ± 10.48	91.77 ± 10.14	96.90 ± 10.23	98.46 ± 10.06	< 0.001
HbA1c, %	5.31 ± 0.36	5.20 ± 0.34	5.36 ± 0.36	5.43 ± 0.37	< 0.001
Total cholesterol, mg/dL	203.37 ± 42.17	200.96 ± 43.33	204.52 ± 41.55	205.48 ± 42.00	< 0.001
Triglyceride, mg/dL	111.00 [76.00, 163.00]	108.00 [72.00, 162.00]	113.00 [78.00, 163.00]	105.00 [81.00, 147.00]	0.001
HDL-cholesterol, mg/dL	53.66 ± 16.10	55.19 ± 15.72	52.88 ± 16.23	54.11 ± 16.02	< 0.001
eGFR, mL/min/1.73 m^2^	95.85 ± 37.70	109.05 ± 44.81	89.74 ± 31.73	76.90 ± 31.39	< 0.001
Hs-cTnI, ng/L	2.59 [1.16, 5.04]	0.70 [0.09, 1.14]	3.88 [2.55, 6.58]	81.60 [58.73, 140.01]	< 0.001
Hs-cTnT, ng/L	5.18 [3.58, 8.16]	3.58 [2.72, 4.67]	6.34 [4.46, 10.00]	16.90 [10.47, 32.39]	< 0.001

Triglyceride, Hs-cTnI and Hs-cTnT were present as median [Q1, Q3]

*BP* blood pressure, *HDL* high-density lipid, *eGFR* estimated glomerular filtration rate, *Hs* high sensitivity, *cTnI* cardiac Troponin I, *cTnT* cardiac Troponin T, *LOD* limits of detection, *99th URL* 99th percentile upper reference limit

Over a median follow-up period of 17.1 years (IQR, 15.3–18.8 years), there were 2537 all-cause deaths, of which 792 were cardiovascular deaths. Additional file 1: Figure S1 illustrates the survival probability among participants with different Hs-cTn concentrations. Higher concentrations of both Hs-cTnI and Hs-cTnT were associated with higher risks of all-cause and cardiovascular mortality (All log-rank P-values < 0.001).

Table 2 presents the association between Hs-cTn concentration and all-cause and cardiovascular mortality. After adjusting for all the confounders, compared with Hs-cTnI concentration < LOD, the HRs (95% CIs) for all-cause mortality were 1.08 (0.94, 1.25) and 1.74 (1.35, 2.24) in Hs-cTnI concentration between LOD to 99th URL and Hs-cTnI concentration ≥ 99th URL, respectively. The corresponding HRs (95% CIs) for cardiovascular mortality were 1.12 (0.83, 1.49) and 2.10 (1.36, 3.24). Compared to Hs-cTnT concentration < LOD, the HRs (95% CIs) for all-cause mortality were 1.07 (0.81, 1.41) and 2.07 (1.53, 2.81) in Hs-cTnT concentration between LOD to 99th URL and Hs-cTnT concentration ≥ 99th URL, respectively. The corresponding HRs (95% CIs) for cardiovascular mortality were 1.27 (0.66, 2.43) and 2.92 (1.47, 5.80). Similar results were observed for the relationship between Hs-cTnI (A) and mortality and between Hs-cTnI (O) and mortality, respectively (Additional file 1: Table S2).

**Table 2:** Association between Hs-cTn concentration and all-cause and cardiovascular mortality

	Hazard ratio (95% confidence interval)			P-trend
	< LOD	LOD–99th URL	≥ 99th URL	
All-cause mortality				
Hs-cTnI				
Model 1	Ref	1.17 (1.02, 1.34)	1.98 (1.57, 2.50)	< 0.001
Model 2	Ref	1.08 (0.94, 1.25)	1.74 (1.35, 2.24)	< 0.001
Hs-cTnT				
Model 1	Ref	1.09 (0.83, 1.42)	2.37 (1.77, 3.15)	< 0.001
Model 2	Ref	1.07 (0.81, 1.41)	2.07 (1.53, 2.81)	< 0.001
Cardiovascular mortality				
Hs-cTnI				
Model 1	Ref	1.24 (0.94, 1.64)	2.92 (1.98, 4.32)	< 0.001
Model 2	Ref	1.12 (0.83, 1.49)	2.10 (1.36, 3.24)	< 0.001
Hs-cTnT				
Model 1	Ref	1.31 (0.71, 2.44)	3.71 (1.94, 7.08)	< 0.001
Model 2	Ref	1.27 (0.66, 2.43)	2.92 (1.47, 5.80)	< 0.001

Model 1 adjusted for age and sex

Model 2 adjusted for age, sex, race, current smoker, systolic blood pressure, diastolic blood pressure, body mass index, total cholesterol, high-density lipid cholesterol, estimated glomerular filtration rate, prediabetes status, antihypertensive medication use, antiplatelet medication use and statin use

*Hs* high sensitivity, *cTnT* cardiac Troponin T, *cTnI* cardiac Troponin I, *LOD* limits of detection, *99th URL* 99th percentile upper reference limit

**Fig. 1:**
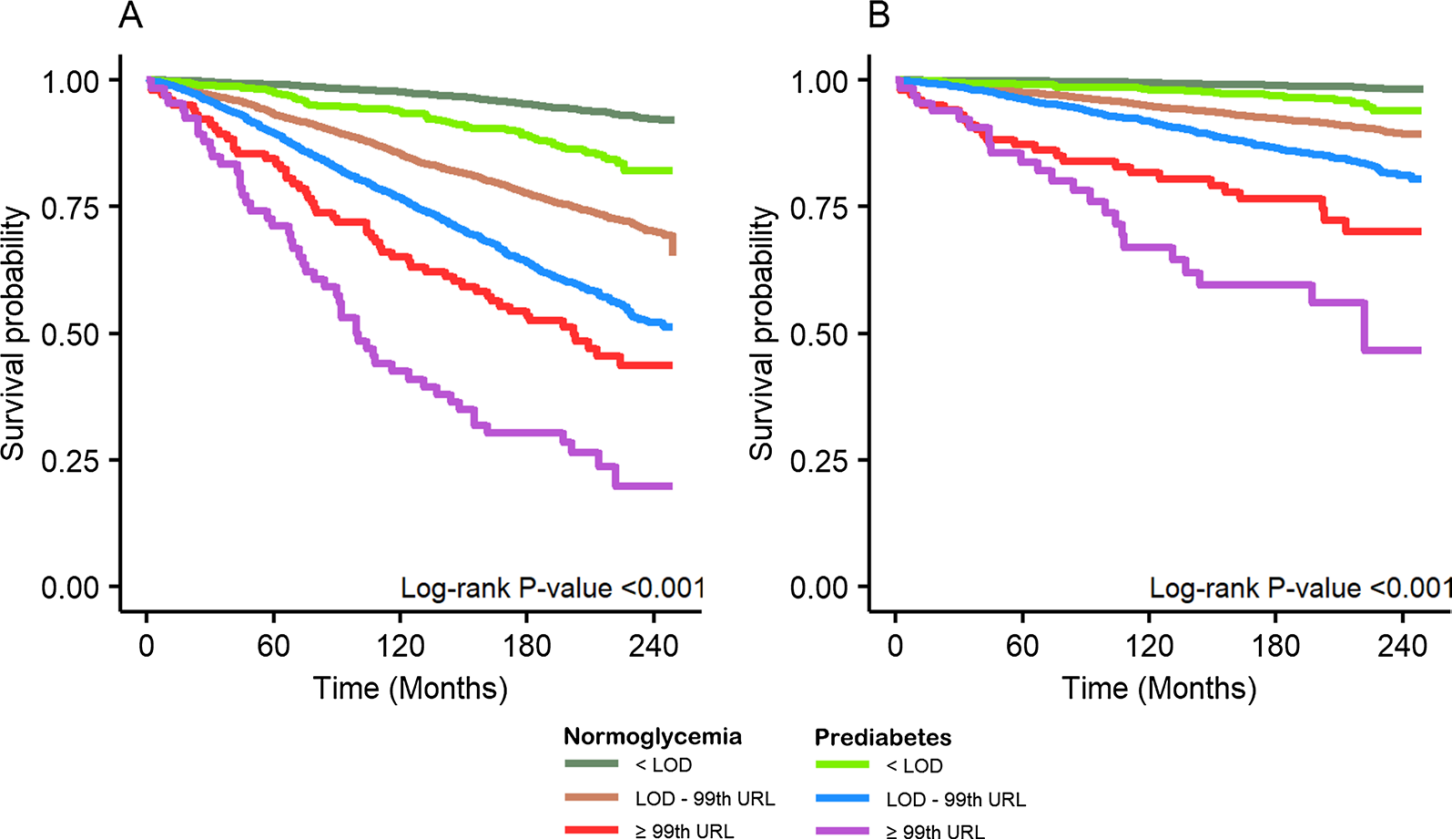
Survival probability of Hs-cTnI concentration stratified by prediabetes status. *Hs-cTnI* high sensitivity cardiac troponin I, *LOD* limits of detection, *99th URL* 99th percentile upper reference limit. **A** All-cause mortality, **B** cardiovascular mortality

Figure 1 displays the survival probability stratified by Hs-cTnI concentrations and prediabetes status. Notably, the K–M curve in the prediabetes subgroup showed a clear separation from that in the normoglycemia subgroup, even when they had Hs-cTnI concentration < LOD. Conversely, the K–M curve in the prediabetes subgroup was close to that in the normoglycemia subgroup, with the same Hs-cTnT concentration (Additional file 1: Fig. S2).

Table 3 shows the association between Hs-cTn concentration and mortality stratified by prediabetes status. A significant interaction between Hs-cTnI and prediabetes on mortality risk was noted (P value for interaction, 0.009 and 0.04 on all-cause and cardiovascular mortality, respectively). The positive relationship between Hs-cTnI concentration and all-cause mortality was observed solely in the prediabetes subgroup [HR (95% CI), 2.27 (1.44, 3.58) for Hs-cTnI concentration ≥ 99th URL, compared to Hs-cTnI concentration < LOD]. In comparison, it was insignificant in the normoglycemia subgroup [HR (95% CI), 1.41 (0.95, 2.10) for Hs-cTnI concentration ≥ 99th URL, compared to Hs-cTnI concentration < LOD]. A comparable trend was found for cardiovascular mortality. Compared with Hs-cTnI concentration < LOD, the corresponding HRs (95% CIs) for Hs-cTnI concentration ≥ 99th URL were 2.55 (1.20, 5.42) in the prediabetes subgroup and 1.72 (0.85, 3.48) in the normoglycemia subgroup. On the contrary, no interaction was found between Hs-cTnT concentration and prediabetes status with all-cause and cardiovascular mortality (P value for interaction > 0.05). Additional file 1: Table S3 presents the interaction analysis for the relationship between the other two Hs-cTnI assays and mortality. The positive relationships between Hs-cTnI (A) and mortality and between Hs-cTnI (O) and mortality remained unchanged in the prediabetes subgroup and normoglycemia subgroup. No interaction was found between Hs-cTnI (A) or Hs-cTnI (O) and prediabetes status with mortality. (all P values for interaction > 0.05).

**Table 3:** Interaction analysis for the relationship between Hs-cTn concentration and mortality

	Hazard ratio (95% confidence interval)			P value for interaction
	< LOD	LOD–99th URL	≥ 99th URL	
All-cause mortality				
Hs-cTnI				
Normoglycemia	Ref	1.14 (0.93, 1.40)	1.41 (0.95, 2.10)	0.009
Prediabetes	Ref	0.97 (0.72, 1.29)	2.27 (1.44, 3.58)	
Hs-cTnT				
Normoglycemia	Ref	1.12 (0.78, 1.61)	2.23 (1.48, 3.35)	0.64
Prediabetes	Ref	0.89 (0.45, 1.77)	1.67 (0.82, 3.39)	
Cardiovascular mortality				
Hs-cTnI				
Normoglycemia	Ref	1.28 (0.83, 1.98)	1.72 (0.85, 3.48)	0.04
Prediabetes	Ref	0.87 (0.50, 1.49)	2.55 (1.20, 5.42)	
Hs-cTnT				
Normoglycemia	Ref	1.48 (0.59, 3.73)	3.23 (1.22, 8.56)	0.58
Prediabetes	Ref	0.80 (0.20, 3.18)	1.98 (0.49, 8.02)	

Model adjusted for age, sex, race, current smoker, systolic blood pressure, diastolic blood pressure, body mass index, total cholesterol, high-density lipid cholesterol, estimated glomerular filtration rate, antihypertensive medication use, antiplatelet medication use and statin use

*Hs* high sensitivity, *cTnT* cardiac Troponin T, *cTnI* cardiac Troponin I, *LOD* limits of detection, *99th URL* 99th percentile upper reference limit

**Fig. 2:**
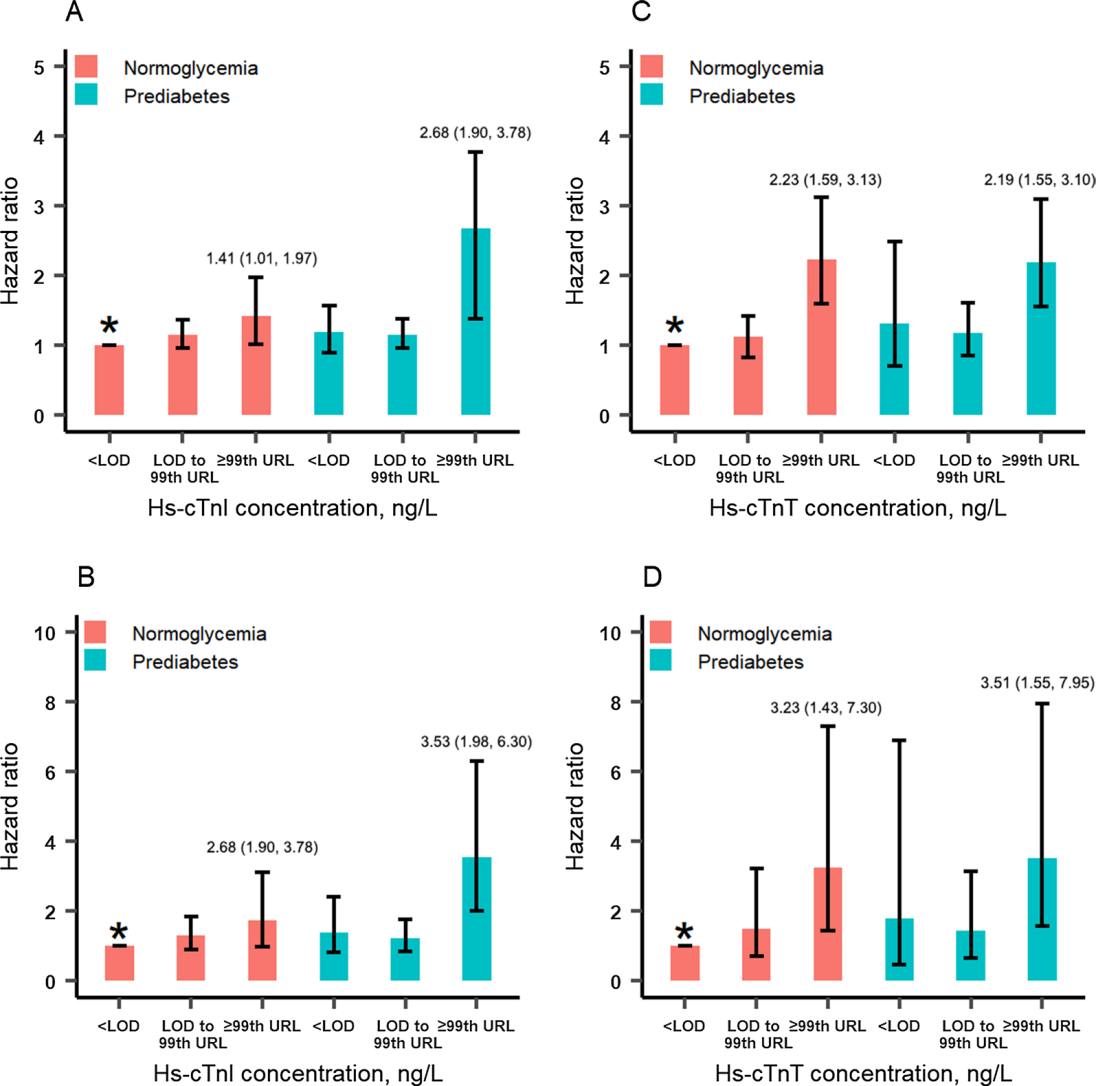
Joint association of Hs-cTn concentration and prediabetes with mortality. *Hs-cTnI* high sensitivity cardiac troponin I, *Hs-cTnT* high sensitivity cardiac troponin T, *LOD* limits of detection, *99th URL* 99th percentile upper reference limit. Adjusted for age, sex, race, current smoker, systolic BP, diastolic BP, body mass index, total cholesterol, high-density lipid cholesterol, eGFR, antihypertensive medication use, antiplatelet medication use, and statin use. **A**, **C** All-cause mortality, **B**, **D** cardiovascular mortality

Figure 2 shows the joint association of Hs-cTn and prediabetes with mortality. Compared to normoglycemia with Hs-cTnI concentration < LOD, the adjusted HRs (95% CIs) for all-cause mortality of participants with Hs-cTnI concentration ≥ 99th URL were 1.41 (1.01, 1.97) in the normoglycemia subgroup and 2.68 (1.90, 3.78) in the prediabetes subgroup (Fig. 2A). The corresponding HRs (95% CIs) were 2.68 (1.90, 3.78) and 3.53 (1.98, 6.30) for cardiovascular mortality (Fig. 2B). HRs (95% CIs) for all-cause mortality of participants with Hs-cTnT concentration ≥ 99th URL was 2.23 (1.59, 3.13) in the normoglycemia subgroup and 2.19 (1.55, 3.10) in the prediabetes subgroup, compared to normoglycemia with Hs-cTnT concentration < LOD (Fig. 2C). The corresponding HRs (95% CIs) were 3.23 (1.43, 7.30) and 3.51 (1.55, 7.95) for cardiovascular mortality (Fig. 2D).

**Fig. 3:**
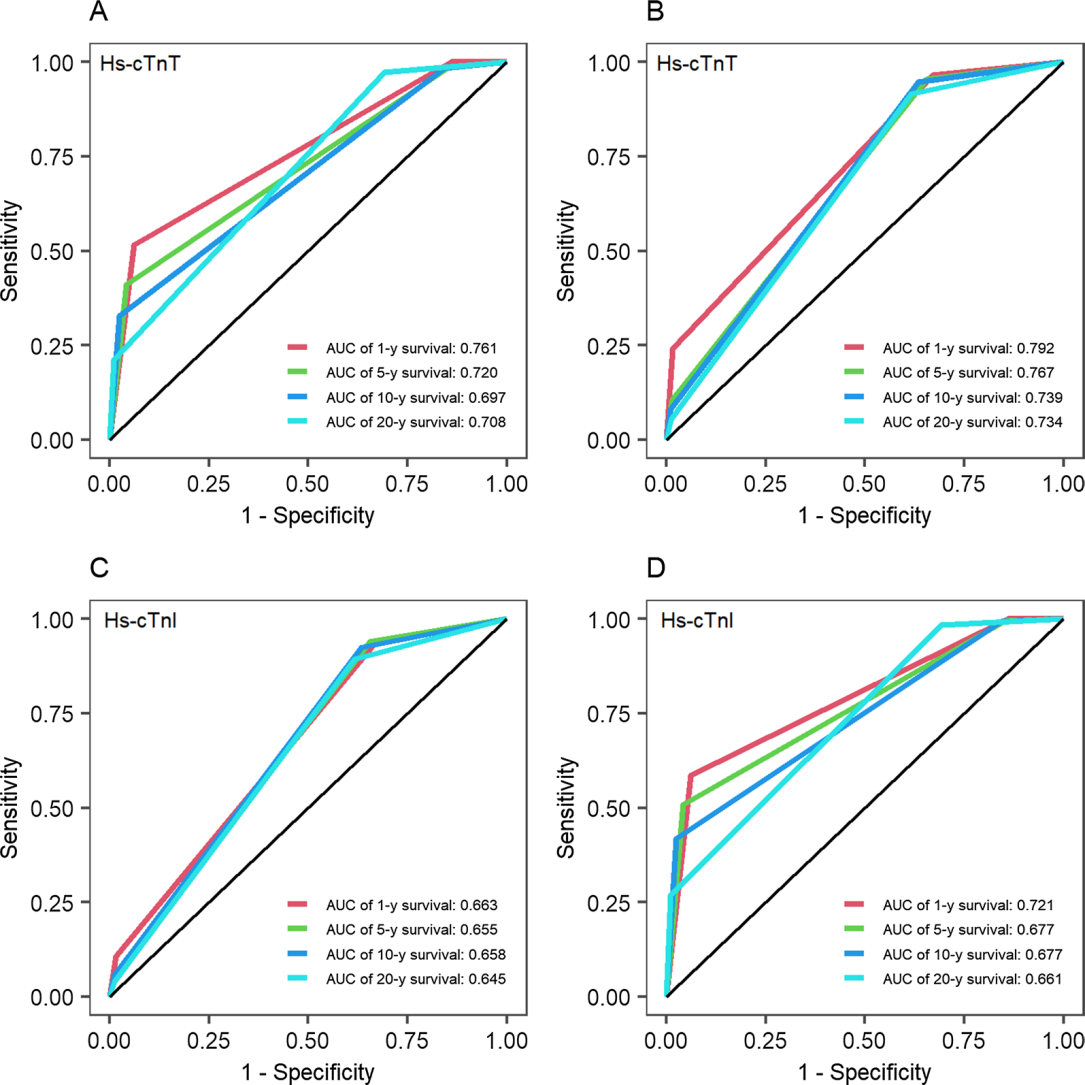
Time-dependent receiver operating characteristics curves for Hs-cTnT and Hs-cTnI. *Hs-cTnI* high sensitivity cardiac troponin I, *Hs-cTnT* high sensitivity cardiac troponin T. **A**, **C** All-cause mortality, **B**, **D** cardiovascular mortality

Figure 3 shows the tROC curves for Hs-cTn in predicting all-cause and cardiovascular mortality. The tAUC values for Hs-cTnT ranged between 0.697 and 0.761 for all-cause mortality and between 0.734 and 0.792 for cardiovascular mortality, depending on the duration of follow-up. The corresponding tAUC values for Hs-cTnI were in the range of 0.645 to 0.663 and 0.661 to 0.721 for all-cause and cardiovascular mortality, respectively.

As presented in Table 4, compared to the based model that includes Framingham risk factors, the NRI (95% CI) for adding Hs-cTnT was 0.25 (0.21, 0.27) for all-cause mortality and 0.33 (0.26, 0.39) for cardiovascular mortality. The corresponding NRI (95% CI) for Hs-cTnI were 0.04 (0, 0.06) and 0.07 (0.01, 0.13).

**Table 4:** Net reclassification improvement for Hs-cTn

	NRI (95% CI) for adding Hs-cTnI	
	Standard model^a^	Updated model^b^
All-cause mortality	Ref	0.04 (0, 0.06)
Cardiovascular mortality	Ref	0.07 (0.01, 0.13)

	NRI (95% CI) for adding Hs-cTnT	
	Standard model^a^	Updated model^c^
All-cause mortality	Ref	0.25 (0.21, 0.27)
Cardiovascular mortality	Ref	0.33 (0.26, 0.39)

	NRI (95% CI) for comparing Hs-cTnT and Hs-cTnI	
	Standard model^b^	Updated model^c^
All-cause mortality	Ref	0.22 (0.19, 0.25)
Cardiovascular mortality	Ref	0.29 (0.21, 0.37)

*FRF* Framingham risk factors, including age, sex, smoking, systolic blood pressure, total cholesterol, high density lipid cholesterol, and anti-hypertensive medication, *NRI* Net reclassification improvement, *CI* confidence interval

^a^Model with FRF

^b^Model with FRF + Hs-cTnI

^c^Model with FRF + Hs-cTnT

## Discussion

In this large prospective cohort study of U.S. adults without diabetes, we identified three significant findings. Firstly, elevation in the blood level of Hs-cTnI or Hs-cTnT was significantly associated with increased all-cause and cardiovascular mortality, independently of traditional risk factors. Secondly, we found a significant interaction between prediabetes and Hs-cTnI, but not Hs-cTnT, on all-cause and cardiovascular mortality. Specifically, we observed a positive relationship between Hs-cTnI and mortality only in the prediabetic subgroup but not in normoglycemic individuals. In contrast, the positive relationship between Hs-cTnT and mortality was observed in both the prediabetic and normoglycemic subgroups. Lastly, Hs-cTnT showed better predictive performance than Hs-cTnI. Our findings suggest that Hs-cTnT may be a more useful biomarker for risk assessment in the non-diabetic population.

Our study confirmed the positive association of Hs-cTnT and Hs-cTnI with all-cause and cardiovascular mortality in non-diabetic individuals without previous cardiovascular disease, which is consistent with previous studies. These studies have shown that elevations of Hs-cTnI and Hs-cTnT were associated with a higher risk of cardiovascular events and mortality in cardiovascular patients and the general population [14–19]. Only a few studies examined this relationship in the non-diabetic population. In the post hoc analysis of the Systolic Blood Pressure Intervention Trial (SPRINT), a US trial comparing blood pressure targets in 9361 persons without diabetes, elevated baseline level of Hs-cTnT was found positively associated with all-cause mortality, heart failure, and composite adverse severe events [20, 21]. However, analyzing data from The Women’s Health Study, Everett et al. found that in women without diabetes, elevated Hs-cTnT was not significantly associated with cardiovascular death, myocardial infarction, stroke, and their combination [22].

Our study revealed that even in individuals with normal Hs-cTn concentrations, those with prediabetes were at a higher risk of mortality, and the risk increased with Hs-cTn concentration. Prediabetes is a known toxic cardiometabolic state associated with microvascular and macrovascular complications [23] and is characterized by weight gain, insulin resistance, and beta-cell dysfunction [1]. Various diabetes-related pathophysiological defects, including endothelial dysfunction [24], arterial stiffness [25], and increased lipolysis [26], are also observed in prediabetic subjects. These metabolic disorders further caused progressive macrovascular complications and repeated subclinical myocardial injury, thereby elevating the blood concentration of cTn [27]. In the Atherosclerosis Risk in Communities (ARIC) study involving 8153 participants without known diabetes or cardiovascular disease, Whelton et al. found that participants with elevated hs-cTnT levels at baseline had a higher incidence of diabetes [28]. It reinforces the idea that by the time people develop diabetes, atherosclerotic cardiovascular disease has already started to develop and can be detected by Hs-cTnT. Our study indicated that the risk of cardiovascular disease was even higher when both elevated Hs-cTn and prediabetes existed at the same time. More attention is needed on early screening of prediabetes and Hs-cTn biomarkers elevation.

Primary lifestyle modifications and secondary antidiabetic medication use can significantly reduce the incidence of diabetes among individuals with prediabetes. In addition, these interventions improve other intermediate outcomes such as reduction in blood pressure, body weight, and BMI [3]. Although previous randomized clinical trials of intensive glucose lowering in type 2 diabetes found no strong evidence of cardiovascular benefit, there are insufficient data to assess the advantage of early screening and intervention for prediabetes [29]. Our study addresses this knowledge gap by revealing possible early-stage myocardial injury in patients with prediabetes. They may benefit from statin therapy, reducing the Hs-cTnI concentration and lowering the coronary disease risk [30]. Further trials are needed to verify the benefit of other primary interventions, such as antiplatelet therapy, for cardiovascular disease in individuals with prediabetes.

### Strengths and limitations

The present study has several strengths. Firstly, NHANES is a well-designed prospective study with a large sample size. Secondly, we compared the associations of two common biomarkers, Hs-cTnI and Hs-cTnI, with the hard outcomes of cardiovascular and all-cause mortality. Thirdly, we revealed the interaction effect of prediabetes, providing clues for further mediation analysis in dysglycemia-related cardiovascular disease.

Several limitations should be considered as well. First, we only used the ADA criteria in the prediabetes definition, although there are other cutoffs to define prediabetes [31]. Second, Hs-cTnI and Hs-cTnT were only measured once in the study, so a longitudinal association between Hs-cTn and mortality could not be demonstrated. Third, some potential confounders, such as changes in dietary habits, need to be addressed. Fourth, the causality between elevated Hs-cTnI and prediabetes cannot be determined due to the observational study design. Nevertheless, early detection of Hs-cTn may be beneficial regardless of the etiology, even if the management is confined to modification of lifestyle changes and cardiovascular risk factors. Our study adds to the growing evidence base supporting the use of cardiac troponins as a biomarker in the clinical setting.

## Conclusion

In conclusion, elevations in Hs-cTnI and Hs-cTnT are associated with a higher risk of all-cause and cardiovascular mortality in the non-diabetic population. The robust association of Hs-cTnT with mortality in prediabetic and normoglycemic subgroups highlights its potential to identify individuals with greater risks and facilitates early interventions to prevent cardiovascular diseases.

### Supplementary Information

**Additional file 1: Table S1.** Baseline characteristics according to Hs-cTnT concentration. **Table S2.** Association between Hs-cTnI concentration and all-cause and cardiovascular mortality. **Table S3.** Interaction analysis for the relationship between Hs-cTnI concentration and mortality. **Figure S1.** Survival probability according to Hs-cTn concentration. **Figure S2.** Survival probability according to Hs-cTnT concentration stratified by prediabetes status.

## Data Availability

The datasets generated and analyzed during the current study are available on the NHANES website: https://www.cdc.gov/nchs/nhanes/index.htm.
