# Supplementary material for: Association between high sensitivity cardiac troponin and mortality risk in the non-diabetic population: findings from the National Health and Nutrition Examination Survey
Source: Cardiovasc Diabetol. 2023 Oct 30;22:296. doi: 10.1186/s12933-023-02003-2 (PMC10617237; doi:10.1186/s12933-023-02003-2)
Supplement: Supplementary file 1 — Additional file 1: Table S1. Baseline characteristics according to Hs-cTnT concentration. Table S2. Association between Hs-cTnI concentration and all-cause and cardiovascular mortality. Table S3. Interaction analysis for the relationship between Hs-cTnI concentration and mortality. Figure S1. Survival probability according to Hs-cTn concentration. Figure S2. Survival probability according to Hs-cTnT concentration stratified by prediabetes status. [file 12933_2023_2003_MOESM1_ESM.docx]

**Table S1. Baseline characteristics according to Hs-cTnT concentration**

|  | < LOD | LOD to 99^th^ URL | ≥ 99^th^ URL | P-value |  |
| --- | --- | --- | --- | --- | --- |
|  | 1455 | 8265 | 673 |  |  |
| Age, year | 33.11 ± 9.75 | 48.49 ± 18.12 | 75.04 ± 12.61 | <0.001 |  |
| Female (%) | 1296 (89.1) | 3871 (46.8) | 370 (55.0) | <0.001 |  |
| Race and ethnicity (%) |  |  |  | <0.001 |  |
| Hispanic | 422 (29.0) | 2229 (27.0) | 80 (11.9) |  |  |
| Non-Hispanic White | 706 (48.5) | 4343 (52.5) | 473 (70.3) |  |  |
| Non-Hispanic Black | 270 (18.6) | 1410 (17.1) | 104 (15.5) |  |  |
| others | 57 (3.9) | 283 (3.4) | 16 (2.4) |  |  |
| Current smoker (%) | 344 (23.6) | 1871 (22.6) | 95 (14.1) | <0.001 |  |
| Alcohol drinker (%) | 854 (58.7) | 5495 (66.5) | 369 (54.8) | <0.001 |  |
| Systolic BP, mmHg | 112.12 ± 12.88 | 125.84 ± 20.18 | 143.80 ± 27.61 | <0.001 |  |
| Diastolic BP, mmHg | 67.23 ± 12.40 | 71.54 ± 13.28 | 65.35 ± 19.82 | <0.001 |  |
| Body mass index, kg/m² | 27.72 ± 6.43 | 28.02 ± 5.91 | 27.16 ± 5.69 | 0.001 |  |
| Prediabetes (%) | 141 (9.7) | 2249 (27.2) | 291 (43.2) | <0.001 |  |
| Hypertension (%) | 109 (7.5) | 2671 (32.3) | 476 (70.7) | <0.001 |  |
| Antihypertensive medication (%) | 60 (4.1) | 1462 (17.7) | 340 (50.5) | <0.001 |  |
| Statin (%) | 9 (0.6) | 716 (8.7) | 120 (17.8) | <0.001 |  |
| Antiplatelet medication (%) | 0 (0.0) | 63 (0.8) | 39 (5.8) | <0.001 |  |
| Fasting blood glucose, mg/dL | 89.68 ± 9.21 | 95.96 ± 10.33 | 98.72 ± 10.85 | <0.001 |  |
| HbA1c, % | 5.13 ± 0.32 | 5.33 ± 0.35 | 5.46 ± 0.35 | <0.001 |  |
| Total cholesterol, mg/dL | 196.19 ± 42.25 | 204.44 ± 41.60 | 205.81 ± 47.21 | <0.001 |  |
| Triglyceride, mg/dL | 98.00 [69.00, 149.00] | 113.00 [77.00, 165.00] | 114.00 [83.75, 157.00] | <0.001 |  |
| HDL-Cholesterol, mg/dL | 55.73 ± 15.63 | 53.12 ± 15.93 | 55.74 ± 18.43 | <0.001 |  |
| eGFR, mL/min/1.73m2 | 125.51 ± 46.50 | 93.32 ± 33.09 | 62.90 ± 27.57 | <0.001 |  |
| Hs-cTnT, ng/L | 2.12 [1.68, 2.60] | 5.48 [4.08, 7.89] | 25.10 [18.76, 34.23] | <0.001 |  |
| Hs-cTnI, ng/L | 0.93 [0.15, 1.93] | 2.74 [1.38, 4.95] | 10.80 [5.70, 23.54] | <0.001 |  |
| BP, blood pressure, HDL, high-density lipid, eGFR, estimated glomerular filtration rate,  Hs, high sensitivity, cTnI, cardiac Troponin I, cTnT, cardiac Troponin T, LOD, limits of detection, 99^th^ URL, 99^th^ percentile upper reference limit. Triglyceride, Hs-cTnI and Hs-cTnT were present as median [Q1, Q3] | | | | |  |
|  |  |  |  |  |  |
|  |  |  |  |  |  |
|  |  |  |  |  |  |

**Table S2. Association between Hs-cTnI concentration and all-cause and cardiovascular mortality**

|  |  | Hazard ratio (95% confidence interval) | | |  |  |
| --- | --- | --- | --- | --- | --- | --- |
| All-cause mortality | | | | | |  |
|  |  | < LOD | LOD - 99^th^ URL | ≥ 99^th^ URL | P-trend |  |
| Hs-cTnI (A) | Model 1 | ref | 1.41 (1.25, 1.60) | 2.66 (2.11, 3.36) | <0.001 |  |
|  | Model 2 | ref | 1.34 (1.17, 1.53) | 2.12 (1.63, 2.77) | <0.001 |  |
|  |  |  |  |  |  |  |
| Hs-cTnI (O) | Model 1 | ref | 1.32 (1.17, 1.49) | 3.17 (2.60, 3.86) | <0.001 |  |
|  | Model 2 | ref | 1.27 (1.12, 1.44) | 2.61 (2.09, 3.26) | <0.001 |  |
|  |  |  |  |  |  |  |
|  |  |  |  |  |  |  |
| Cardiovascular mortality | | | | | |  |
|  |  | < LOD | LOD - 99^th^ URL | ≥ 99^th^ URL | P-trend |  |
| Hs-cTnI (A) | Model 1 | ref | 1.57 (1.23, 2.02) | 4.69 (3.25, 6.76) | <0.001 |  |
|  | Model 2 | ref | 1.47 (1.13, 1.92) | 3.35 (2.21, 5.09) | <0.001 |  |
|  |  |  |  |  |  |  |
| Hs-cTnI (O) | Model 1 | ref | 1.76 (1.37, 2.27) | 6.43 (4.59, 9.00) | <0.001 |  |
|  | Model 2 | ref | 1.63 (1.25, 2.12) | 4.69 (3.24, 6.8) | <0.001 |  |
| Hs-cTnI (A), high-sensitivity cardiac Troponin I tested by the Abbott ARCHITECT i2000SR Hs-cTnI (O), high-sensitivity cardiac Troponin I tested by the Ortho Vitros 3600 LOD, limits of detection, 99^th^ URL, 99th percentile upper reference limit. Model 1 adjusted for age and sex. Model 2 adjusted for age, sex, race, current smoker, systolic blood pressure, diastolic blood pressure, body mass index, total cholesterol, high-density lipid cholesterol, estimated glomerular filtration rate, prediabetes status, antihypertensive medication use, antiplatelet medication use and statin use. | | | | | |  |
|  |  |  |  |  |  |  |
|  |  |  |  |  |  |  |
|  |  |  |  |  |  |  |
|  |  |  |  |  |  |  |
|  |  |  |  |  |  |  |
|  |  |  |  |  |  |  |

**Table S3. Interaction analysis for the relationship between Hs-cTnI concentration and mortality**

|  |  | Hazard ratio (95% confidence interval) | | | P-interaction |  |
| --- | --- | --- | --- | --- | --- | --- |
| All-cause mortality | | | | |  |  |
| Hs-cTnI (A) | Normoglycemia | ref | 1.26 (1.05, 1.51) | 2.23 (1.51, 3.30) | 0.19 |  |
|  | preDM | ref | 1.53 (1.17, 2.01) | 2.08 (1.26, 3.42) |  |  |
|  |  |  |  |  |  |  |
| Hs-cTnI (O) | Normoglycemia | ref | 1.28 (1.06, 1.53) | 2.78 (1.99, 3.87) | 0.78 |  |
|  | preDM | ref | 1.26 (0.99, 1.60) | 2.42 (1.63, 3.57) |  |  |
|  |  |  |  |  |  |  |
| Cardiovascular mortality | | | | |  |  |
|  |  | < LOD | LOD - 99^th^ URL | ≥ 99^th^ URL |  |  |
| Hs-cTnI (A) | Normoglycemia | ref | 1.45 (0.98, 2.13) | 3.50 (1.89, 6.50) | 0.9 |  |
|  | preDM | ref | 1.51 (0.90, 2.55) | 3.19 (1.50, 6.79) |  |  |
|  |  |  |  |  |  |  |
| Hs-cTnI (O) | Normoglycemia | ref | 1.85 (1.24, 2.78) | 5.60 (3.18, 9.86) | 0.38 |  |
|  | preDM | ref | 1.34 (0.84, 2.13) | 3.66 (1.95, 6.89) |  |  |
| Hs-cTnI (A), high-sensitivity cardiac Troponin I tested by the Abbott ARCHITECT i2000SR Hs-cTnI (O), high-sensitivity cardiac Troponin I tested by the Ortho Vitros 3600 LOD, limits of detection, 99^th^ URL, 99th percentile upper reference limit. model adjusted for age, sex, race, current smoker, systolic blood pressure, diastolic blood pressure, body mass index, total cholesterol, high-density lipid cholesterol, estimated glomerular filtration rate, antihypertensive medication use, antiplatelet medication use and statin use. | | | | | |  |
|  |  |  |  |  |  |  |
|  |  |  |  |  |  |  |
|  |  |  |  |  |  |  |
|  |  |  |  |  |  |  |
|  |  |  |  |  |  |  |

**Figure S1. Survival probability according to Hs-cTn concentration**


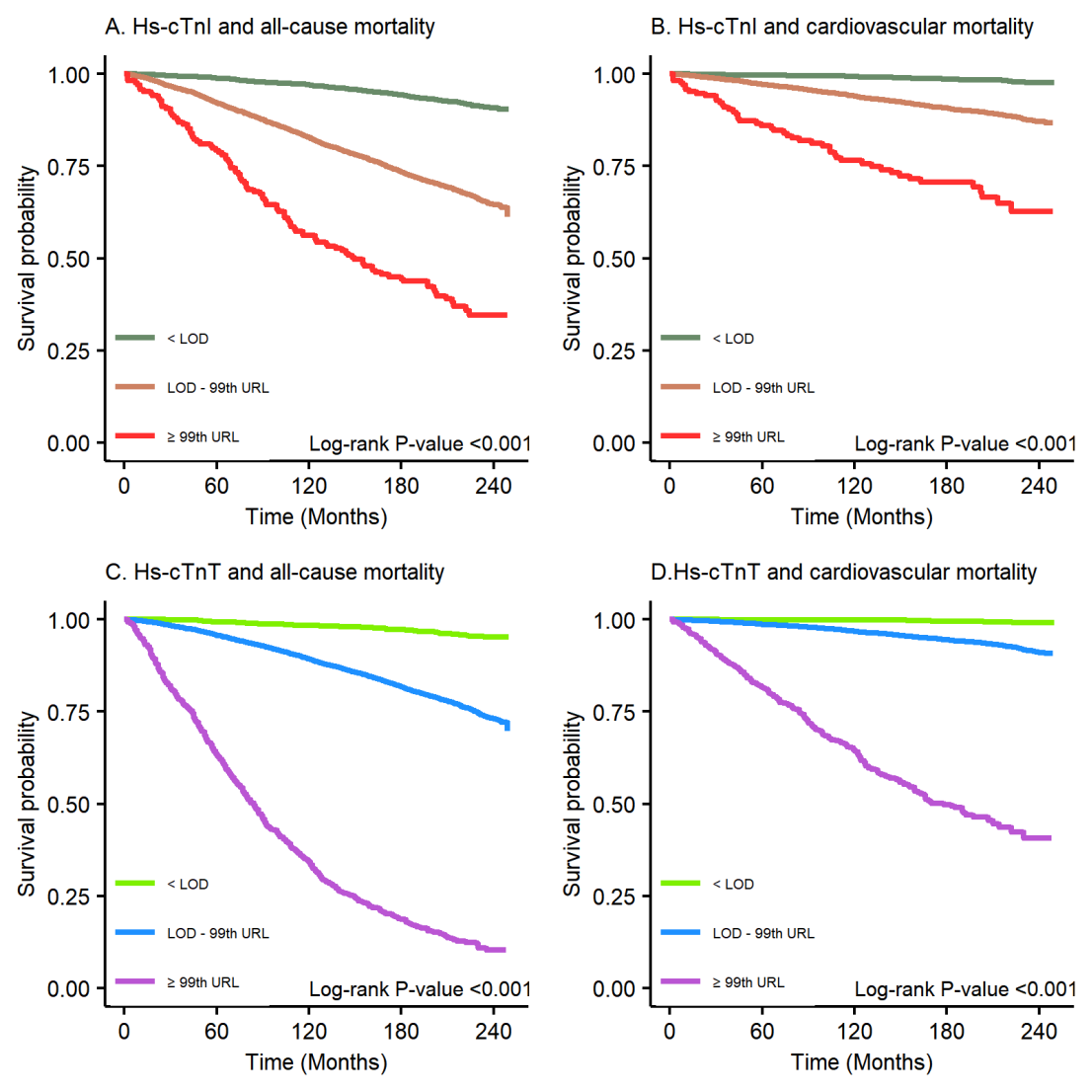


Hs-cTnI, high sensitivity cardiac troponin I.

Hs-cTnT, high sensitivity cardiac troponin T.

LOD, limits of detection, 99^th^ URL, 99^th^ percentile upper reference limit.

**Figure S2. Survival probability according to Hs-cTnT concentration stratified by prediabetes status**


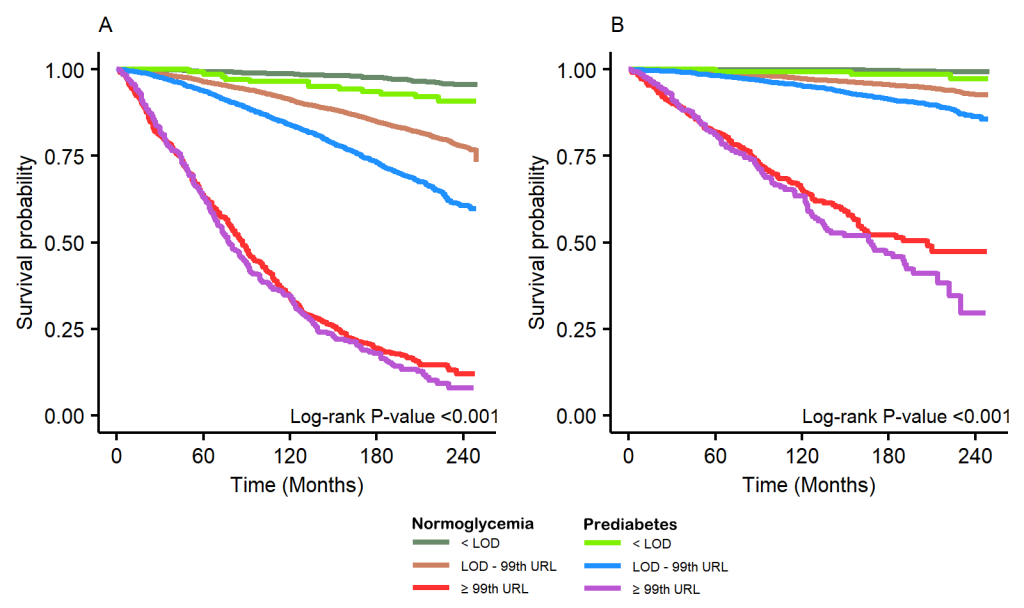


Hs-cTnT, high sensitivity cardiac troponin T, LOD, limits of detection, 99^th^ URL, 99^th^ percentile upper reference limit.

A, all-cause mortality, B, cardiovascular mortality
